# Primary Synovial Sarcoma of External Auditory Canal: A Case Report

**DOI:** 10.7759/cureus.1498

**Published:** 2017-07-20

**Authors:** Aarani Devi, Krishnannair l L Jayakumar

**Affiliations:** 1 Department of Radiation Oncology, Medical College, Trivandrum, Kerala, India

**Keywords:** sarcoma, synovial, ear

## Abstract

Synovial sarcoma is a rare malignant tumor of mesenchymal origin. Primary synovial sarcoma of the ear is extremely rare and to date only two cases have been published in English medical literature. Though the tumor is reported to have an aggressive nature, early diagnosis and treatment may improve the outcome. Here, we report a rare case of synovial sarcoma of the external auditory canal in an 18-year-old male who was managed by chemotherapy and referred for palliation due to tumor progression.

## Introduction

Synovial sarcoma is a malignant tumor of mesenchymal origin with a male to female ratio of 3:2. It occurs most commonly in extremities. Its occurrence in the head and neck region is reported to be uncommon [1]. Jernstrom in 1954 reported the first case of synovial sarcoma of the head and neck [2]. Literature is sparse regarding its occurrence in the ear. Here, we report a rare case of synovial sarcoma of the external auditory canal.

## Case presentation

**Figure 1:**
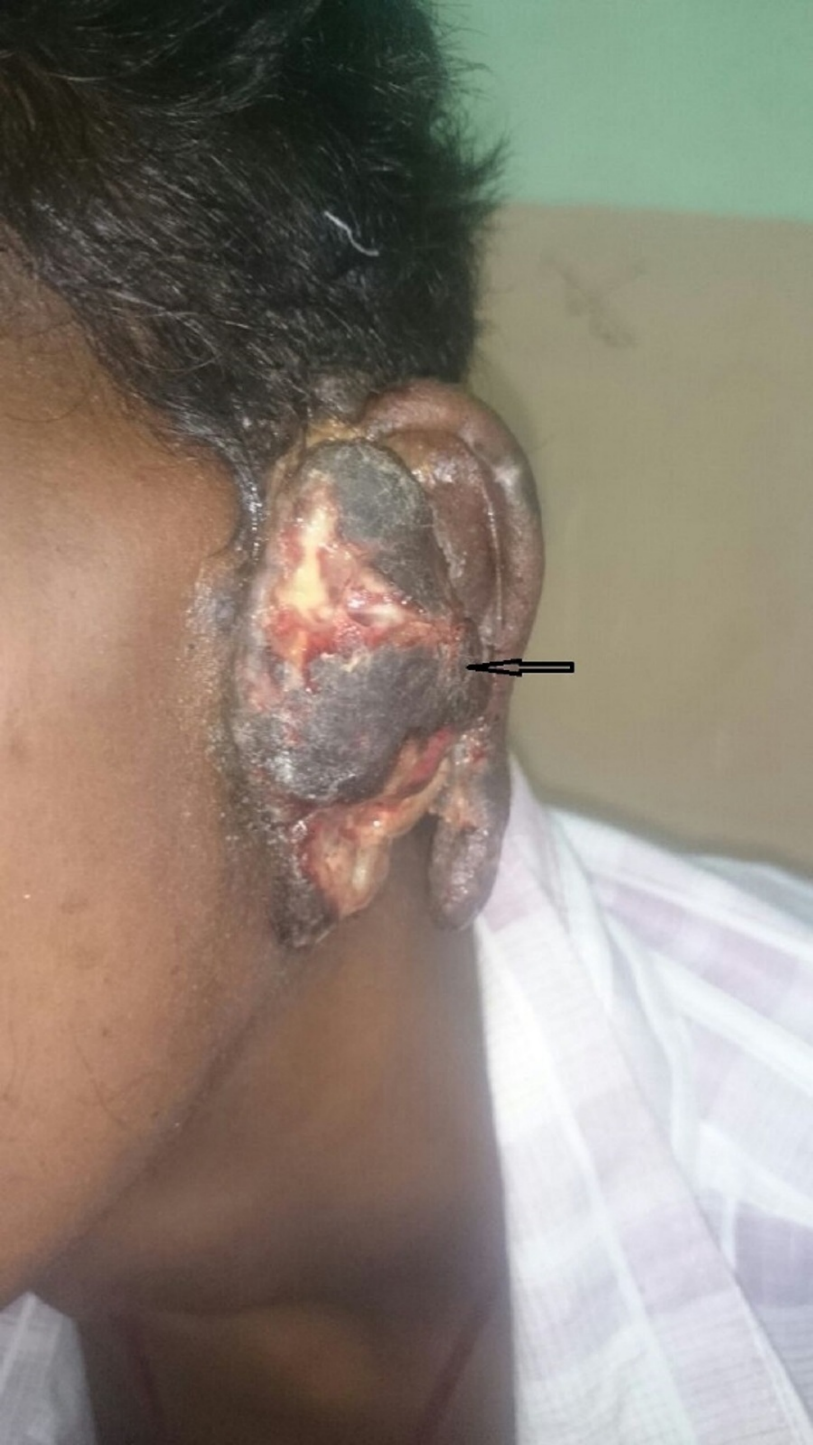
The proliferative lesion with ulceration filling the external auditory canal

An 18-year-old male was referred to the department of radiation oncology with a one-year history of painless progressive swelling over the left pinna. Initial evaluation and excision biopsy from the lesion reported a nonspecific chronic inflammatory lesion with ulceration and granulation tissue formation. The swelling gradually increased in size with associated difficulty in hearing and breathing. A physical examination revealed a proliferative lesion with ulceration filling the external auditory canal (Figure 1).

**Figure 2:**
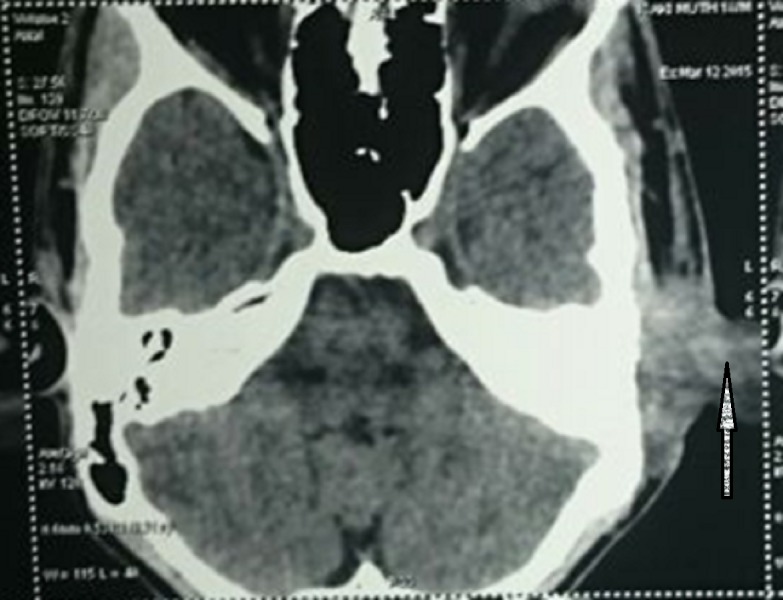
CT of the temporal bone showing a space-occupying lesion at the left post-auricular region CT - Computed tomography.

Computed tomography (CT) of the temporal bone showed a space-occupying lesion measuring 2.4 x 2.1 x 3.4 cm with an ill-defined margin at the left post-auricular region. The lesion was seen extending anteriorly to the base of the pinna and anteromedially to the external auditory canal as well as anteroinferiorly to the left parotid space with minimal erosion of the external auditory canal (Figure 2).

**Figure 3:**
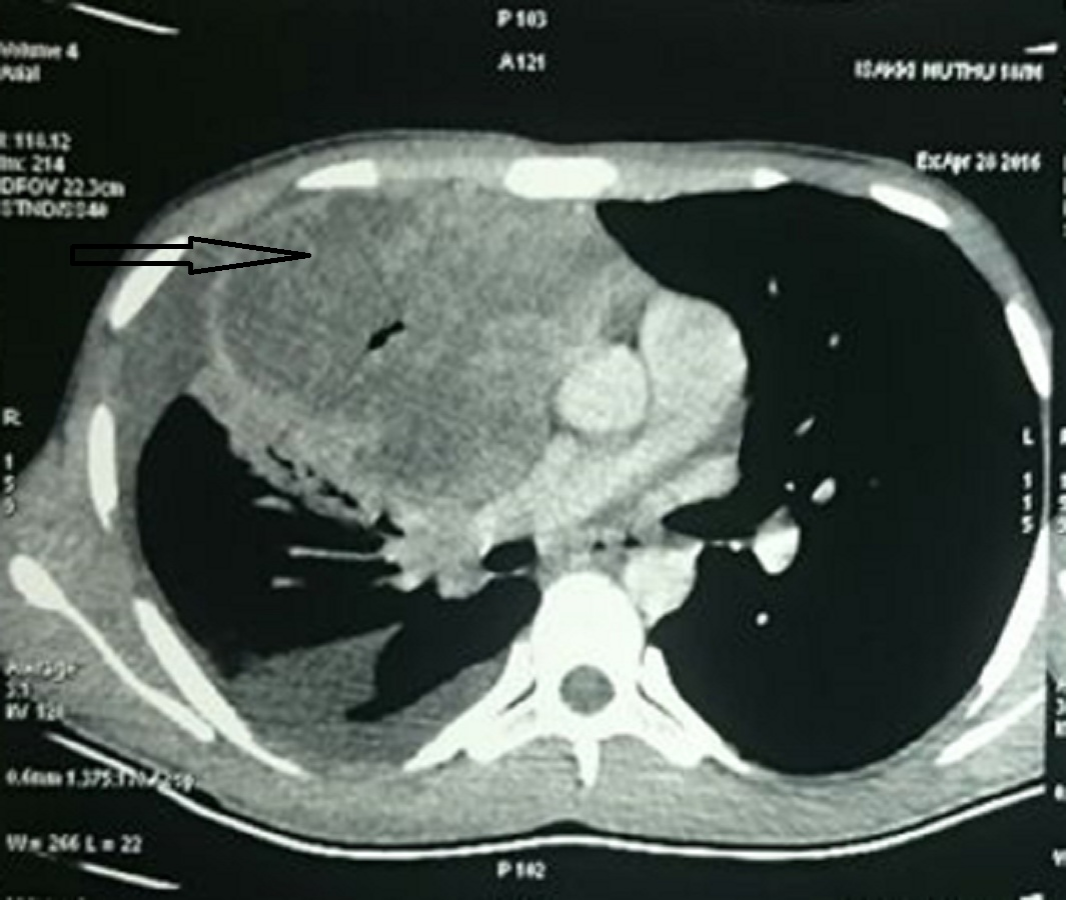
CT of the thorax showing the mass lesion in the upper lobe of the right lung CT - Computed tomography.

A CT of the thorax revealed a mass lesion of size 10.1 x 4.6 x 9.8 cm in the upper lobe of the right lung abutting the pleura, mediastinum, right pulmonary artery, superior vena cava, and ascending aorta (Figure 3).

The biopsy from the ear mass reported a poorly differentiated synovial sarcoma. Immunohistochemistry (IHC) showed cytokeratin (CK), epithelial membrane antigen (EMA), vimentin and CD99 strongly positive, smooth muscle antigen (SMA), CD31 and CD34 negative. A CT-guided biopsy from the lung lesion was also consistent with metastatic deposits from synovial sarcoma. A tumor board was conducted, which concluded that the tumor was inoperable and metastatic and that chemotherapy would be the best option. A chemotherapy schedule consisting of doxorubicin (60 mg/m2 day 1) and ifosfamide (1.5 g/m2 day 1-3) regimen was initiated. A reassessment after three cycles showed disease progression. As the general condition of the patient deteriorated, the tumor board reviewed that the patient could not be taken up for any furthur oncological intervention. The best palliative care was given and the patient expired after two months.

## Discussion

It is estimated that synovial sarcoma accounts for 8-10% of all soft tissue sarcoma. However, synovial sarcoma of the ear is an extremely rare entity. To the best of our knowledge, only one case of primary synovial sarcoma of the middle ear [3] and one case of synovial sarcoma of the inner ear have been reported [4]. Synovial sarcoma initially derived its name from a histologic resemblance to synovial cells, but its actual cellular origin is unknown. There are two morphologic subtypes, monophasic and biphasic. The biphasic subtype has spindle cells resembling synoviocytes and plump epithelial cells forming glands or cords while the monophasic subtype lacks epithelial cells.

In suspected cases, it is essential to consider a CT of the head and neck and a CT of the thorax to define tumor extension and metastasis. Immunohistochemistry studies showed the tumor to be of mesenchymal origin, demonstrating a positive immunoreaction for the epithelial markers, epithelial membrane antigen (EMA) and cytokeratin (CK).

Several factors are reported to determine the prognosis of the tumor of the inner ear. The tumor size and local extent at the time of primary treatment have been found to have an inverse relation to survival. Most metastases originate from hematogenous dissemination and is the most common cause of death [5].

The mainstay of treatment is surgical resection of the primary tumor with negative margin, which is difficult to achieve in the head and neck region. Surgical resection is followed by up to 90% recurrence rate within two years [3]. The studies are controversial regarding pre and postoperative radiotherapy with or without chemotherapy even though this modality of treatment has been reported to improve the survival rates [6].

## Conclusions

Synovial sarcoma of the ear is an extremely rare tumor. Though the tumor has an aggressive nature and poor prognosis, early diagnosis and treatment may improve the outcome and survival. Hence, it is essential that oncologists, otolaryngologists and head and neck surgeons be aware of this rare presentation and take up a multidisciplinary approach toward its management.
